# Supplementary material for: Genetic architecture of a light-temperature coincidence detector
Source: Nat Commun. 2025 Aug 26;16:7947. doi: 10.1038/s41467-025-62194-y (PMC12381198; doi:10.1038/s41467-025-62194-y)
Supplement: Supplementary file 1 — Supplementary Information [file 41467_2025_62194_MOESM1_ESM.pdf]

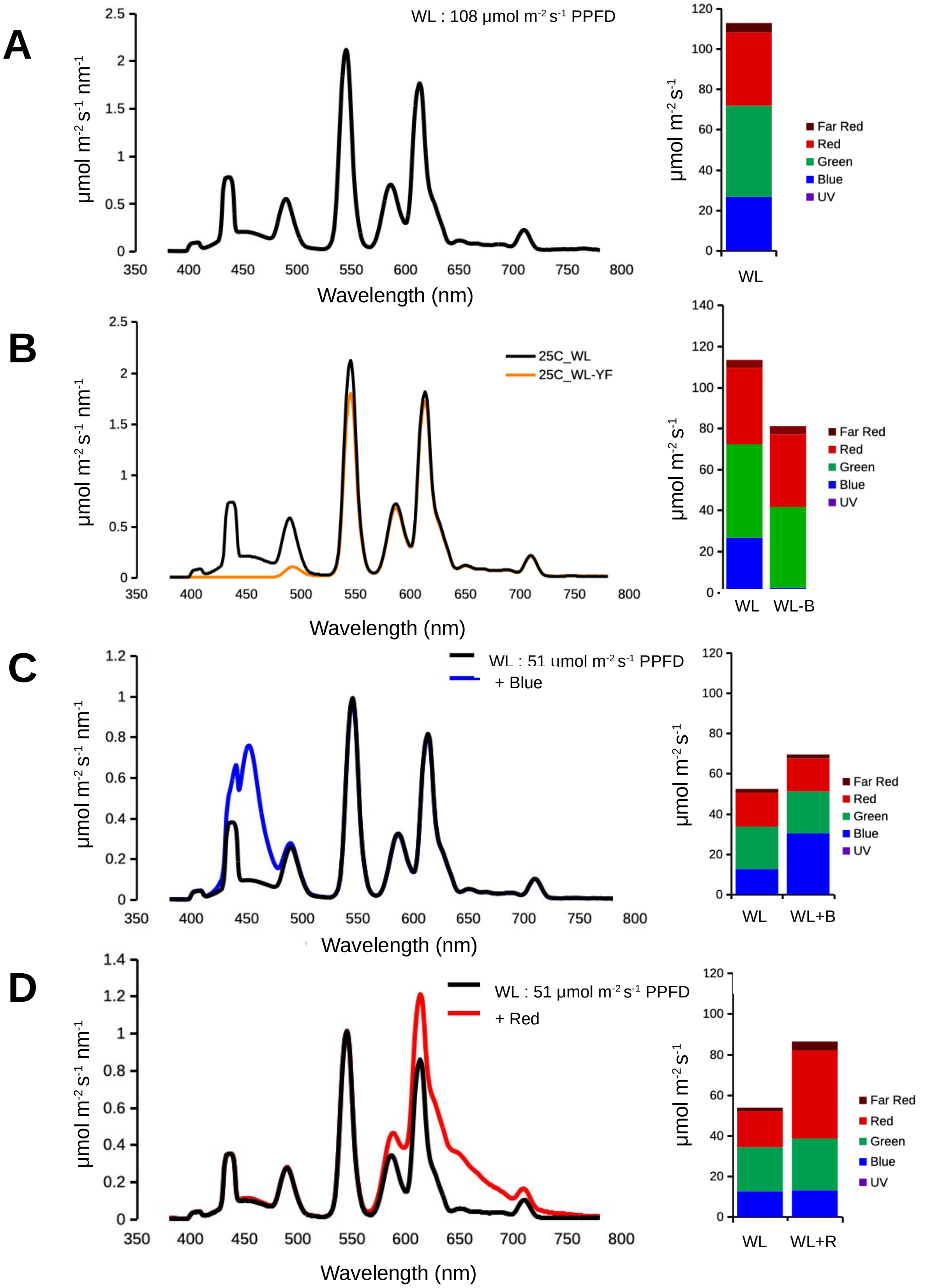

Supplementary Figure 1:
Representative light spectra for flowering and RNAseq experiments.

Bar charts to the right of each spectrum trace shows a breakdown of UV, Blue, Green, Red and Far Red components.
(A) Representative of the light conditions used for most flowering experiments, unless noted, and both RNA sequencing experiments.
(B) White light 105 μmol m^{-2} s^{-1} PPFD vs yellow filtered light.
(C) White light 51 μmol m^{-2} s^{-1} PPFD +/- blue LED supplement.
(D) White light 51 μmol m^{-2} s^{-1} PPFD +/- red LED supplement.

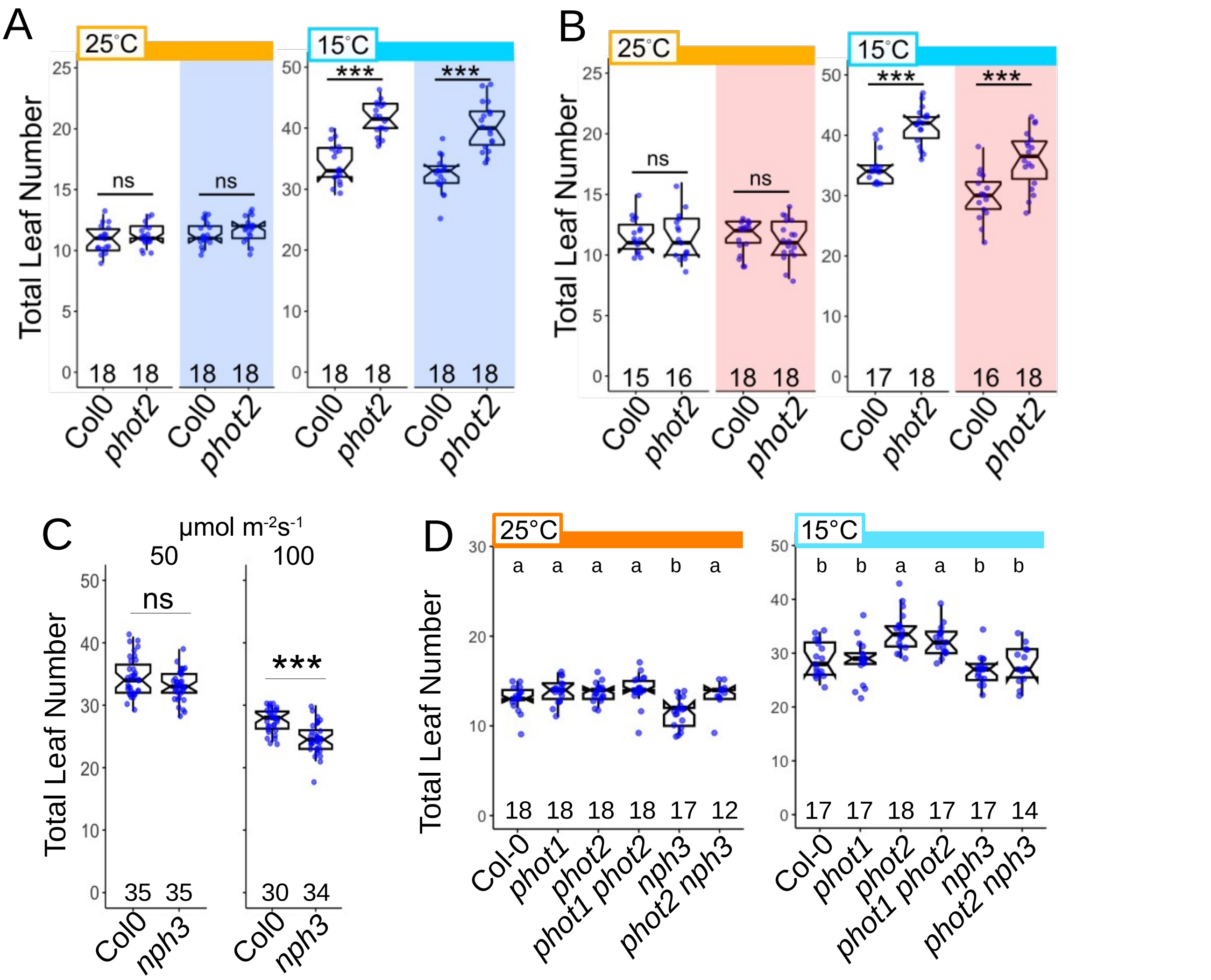

Supplementary Figure 2:
Genetic and Environmental conditions modify TLN at flowering in *phot2* and *nph3*

(A-B) Excess Blue (A) or Red (B) light does not modify the TLN phenotype of *phot2*. Flowering time as assayed by total leaf number at the time of bud appearance in Col-0 and *phot2* under the indicated light and temperature conditions. Colored bars above boxplots indicate temperature: orange = 25˚C, light blue = 15˚C. Light conditions are indicated by background shading: white = white fluorescent light ~50µmol m^{-2}s^{-1}, blue = white fluorescent + blue LED, red = white fluorescent + red LED. Samples were compared by two-way ANOVA (Genotype X Light) + Tukey HSD test. Full statistics are included in Source Data. See Supplementary Figure 1 for spectra. White light data from Col0 and *phot2* in (A) and (B) were combined and plotted in Figure 2D, and further analyzed in Figures 2E and 2F. (C) Light intensity sensitivity in *nph3*. Data for Col-0 and *nph3* under 15˚C are replotted together from Figure 2B (50µmol m^{-2}s^{-1}), and Figure 1A (100µmol m^{-2}s^{-1}). (D) Flowering time under 100µmol m^{-2}s^{-1} for *phot1 phot2* and *phot2 nph3* mutants. Col-0 and *phot2* data are included in the white-light plots in Figure 2A. Alleles: *phot1* (SALK_146058), *phot2* (SALK_142275), *nph3* (SALK_110039). Letters indicate statistically significant differences by one-way ANOVA + Tukey HSD. Boxplots are as in Figure 1.

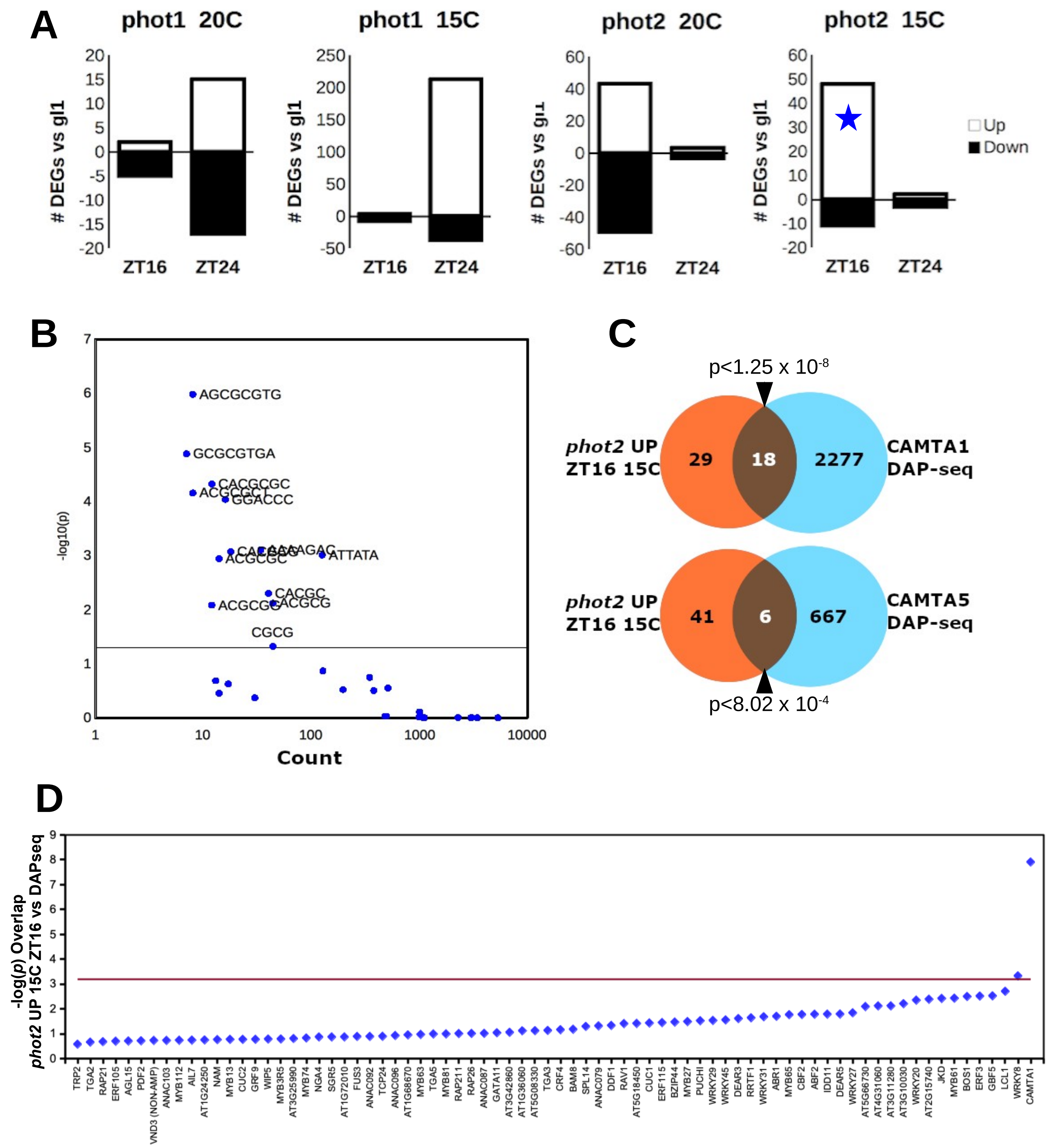

Supplementary Figure 3: RNA sequencing of *PHOT* mutants suggests a connection between the *PHOT2* photoreceptor and *CAMTA* transcription factors

(A) Bar charts show the number of differentially expressed genes (DEGs) identified relative to control (*gl1*) in *phot1-5 gl1* and *phot2-1 gl1* strains grown in soil at either 20˚C or 15˚C, and sampled at ZT16 (end-of-day) or ZT24 (end-of-night) of day 14 in biological duplicate. Blue star indicates the gene set analyzed further in (B-D).
(B) Enrichment of potential cis regulatory elements in 1kb promoter elements of the set of genes up-regulated in *phot2 gl1* in 15˚C, ZT16 calculated using the ELEMENT tool. X-axis is the total number of occurrences of the element in the promoters of the gene set. Y axis is the corrected P-value of the enrichment.
(C) Genes bound by CAMTA1 or CAMTA5 by DAP-seq and the *phot2 gl1* 15˚C ZT16 DEG set. P-value of overlap from hypergeometric test is shown.
(D) Evaluation of DAP-seq target overlap analysis was carried out with 75 other transcription factors in the database with a similar number of reads as CAMTA1 (2000-3750 reads), with each TF represented by a blue diamond and ordered by increasing -log(p) value (hypergeometric test). CAMTA1 is the last and most significantly enriched transcription factor on the plot. Red line is the Bonferroni-corrected threshold for significance.

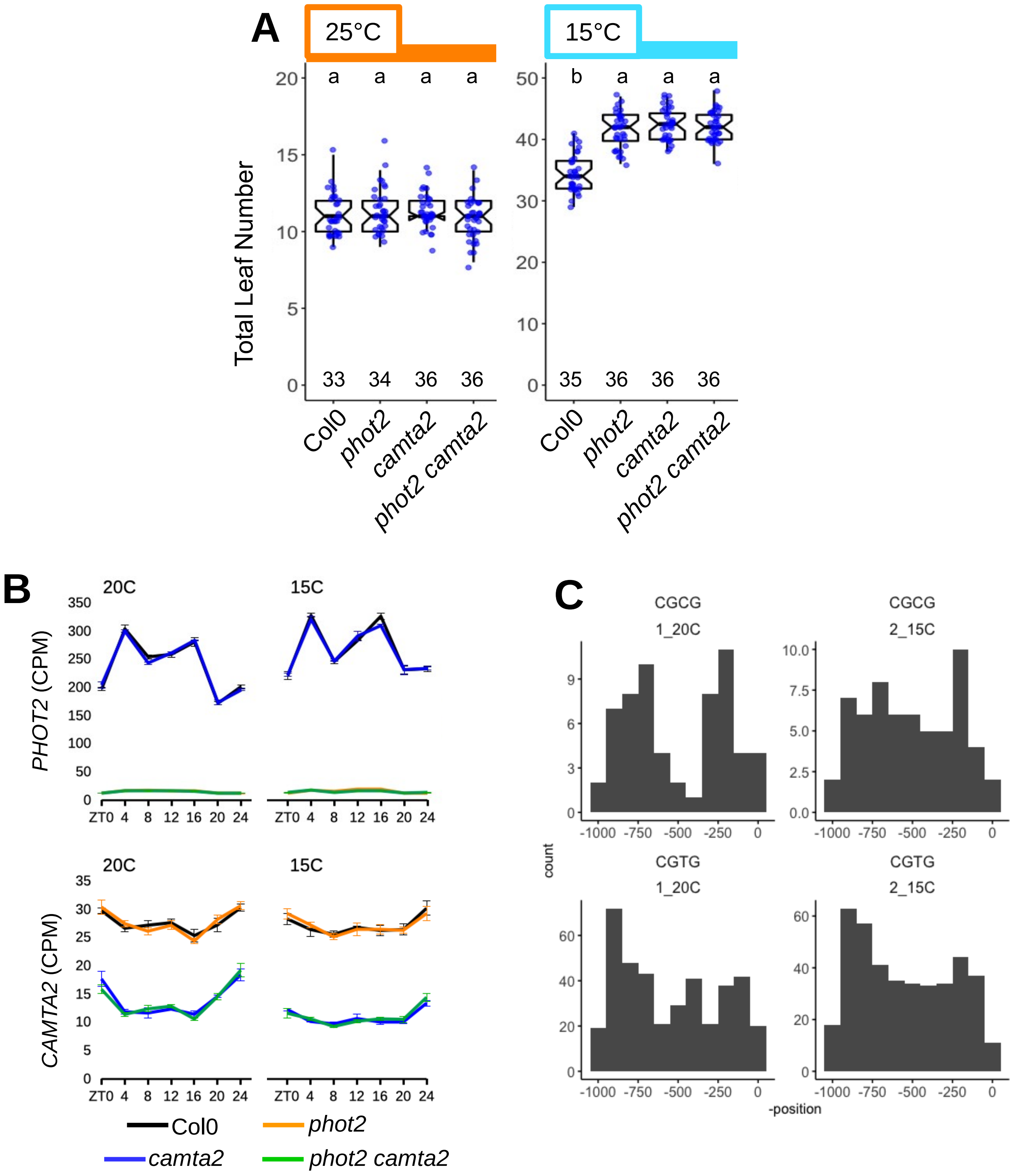

Supplementary Figure 4: Genetic interactions between *phot2* and *camta2*

(A) TLN of Col-0, p*hot2*(SALK_142275), *camta2*(SALK_007027), and *phot2 camta2* plants counted under 50 µmol m^{-2} s^{-1} white light at 25˚C and 15˚C. Col0 and *phot2* data from the same experiment are re-plotted from Supplementary Figure 2B-C (pooled white light) for reference. (B) Counts Per Million reads (CPM) for *PHOT2* and *CAMTA2* transcripts in Col-0, p*hot2*(SALK_142275), *camta2*(SALK_007027), and *phot2 camta2* plants. Error bars are SEM.
(C) Histograms depicting the position of CGCG and CGTG motifs in the promoters of Common DEGs. X axis is in base pairs of the 1kb promoter, with 0 being the transcription start site.

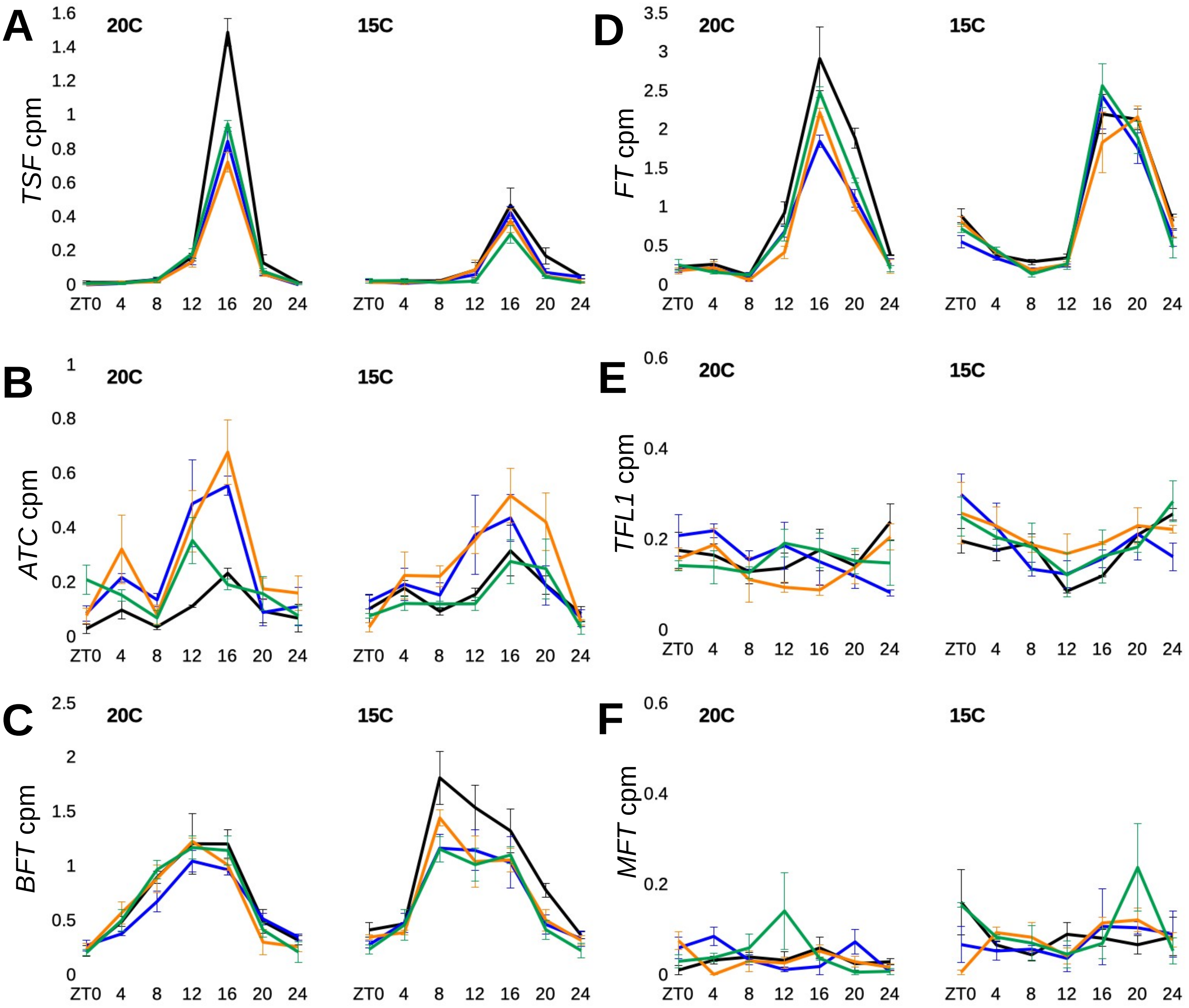

Supplementary Figure 5: Expression of FT-family genes from RNA-seq
Full day expression profiles of the FT-family genes from plants grown at 20˚C or 15˚C derived from RNA sequencing data, expressed in CPM. (A) TSF, (B) ATC, (C) BFT, (D) FT, (E) TFL1, (F) MFT. Traces are Col-0 (black), p*hot2*(SALK_142275)(orange), *camta2-1*(SALK_007027) (blue), and *phot2 camta2* (green).Error bars are SEM. Individual points not shown for clarity.

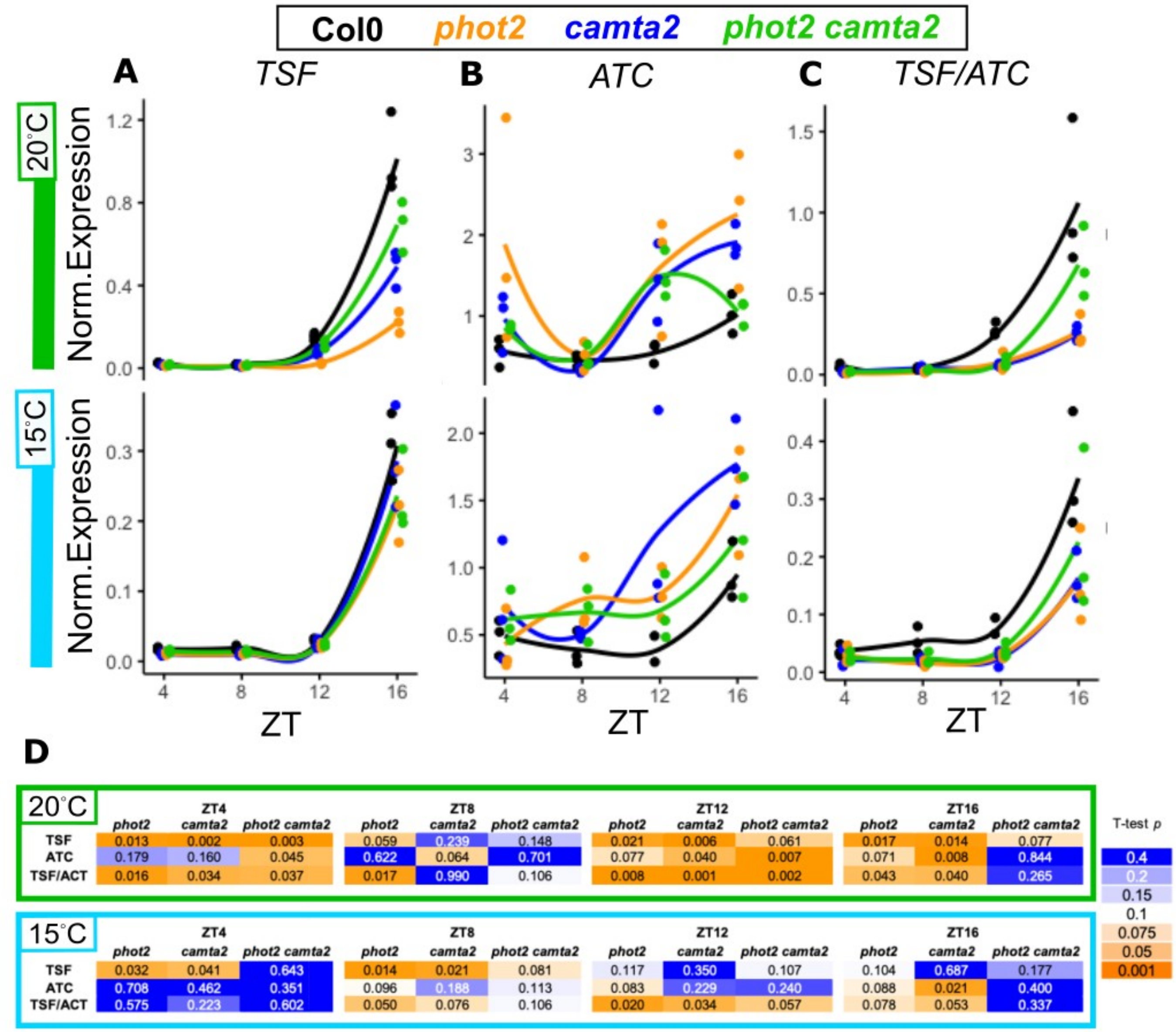

D

20°C	ZT4 phot2	ZT4 camta2	ZT4 phot2 camta2	ZT8 phot2	ZT8 camta2	ZT8 phot2 camta2	ZT12 phot2	ZT12 camta2	ZT12 phot2 camta2	ZT16 phot2	ZT16 camta2	ZT16 phot2 camta2
TSF	0.013	0.002	0.003	0.059	0.239	0.148	0.021	0.006	0.061	0.017	0.014	0.077
ATC	0.179	0.160	0.045	0.622	0.064	0.701	0.077	0.040	0.007	0.071	0.008	0.844
TSF/ACT	0.016	0.034	0.037	0.017	0.990	0.106	0.008	0.001	0.002	0.043	0.040	0.265

15°C	ZT4 phot2	ZT4 camta2	ZT4 phot2 camta2	ZT8 phot2	ZT8 camta2	ZT8 phot2 camta2	ZT12 phot2	ZT12 camta2	ZT12 phot2 camta2	ZT16 phot2	ZT16 camta2	ZT16 phot2 camta2
TSF	0.032	0.041	0.643	0.014	0.021	0.081	0.117	0.350	0.107	0.104	0.687	0.177
ATC	0.708	0.462	0.351	0.096	0.188	0.113	0.083	0.229	0.240	0.088	0.021	0.400
TSF/ACT	0.575	0.223	0.602	0.050	0.076	0.106	0.020	0.034	0.057	0.078	0.053	0.337

T-test *p*
0.4
0.2
0.15
0.1
0.075
0.05
0.001

Supplementary Figure 6:
Florigen-antiflorigen balance is disrupted in *phot2* and *camta2* mutants

(A-B) Quantitative RT-PCR with TaqMan probe detection of TSF (A) and ATC (B) transcripts under 20˚C (green bar) or 15˚C (light blue bar). For each gene, data are normalized such that the mean expression level in Col-0 at ZT16 under 20˚C is set to 1. Reactions were performed in biological triplicate from the RNAseq samples, with the exception of one omitted data point (Col-0, ZT12, 15˚C) as described in Methods. Time points are in Zeitgeber Time (ZT). Traces are mean normalized expression. Col-0 (black), p*hot2*(SALK_142275)(orange), *camta2-1*(SALK_007027) (blue), and *phot2 camta2* (green). Color coding by genotype is shown at top.

(C) TSF/ATC ratio was calculated from the TSF and ATC values in each individual sample and normalized to the mean value of Col-0 ZT16 at 20˚C.

(D) Heatmap of t-test (two-tailed) p-values calculated from data in (A-C). Values in mutant lines were compared to expression in Col-0 within the same time point and temperature. Color coding for the heatmap is at right.

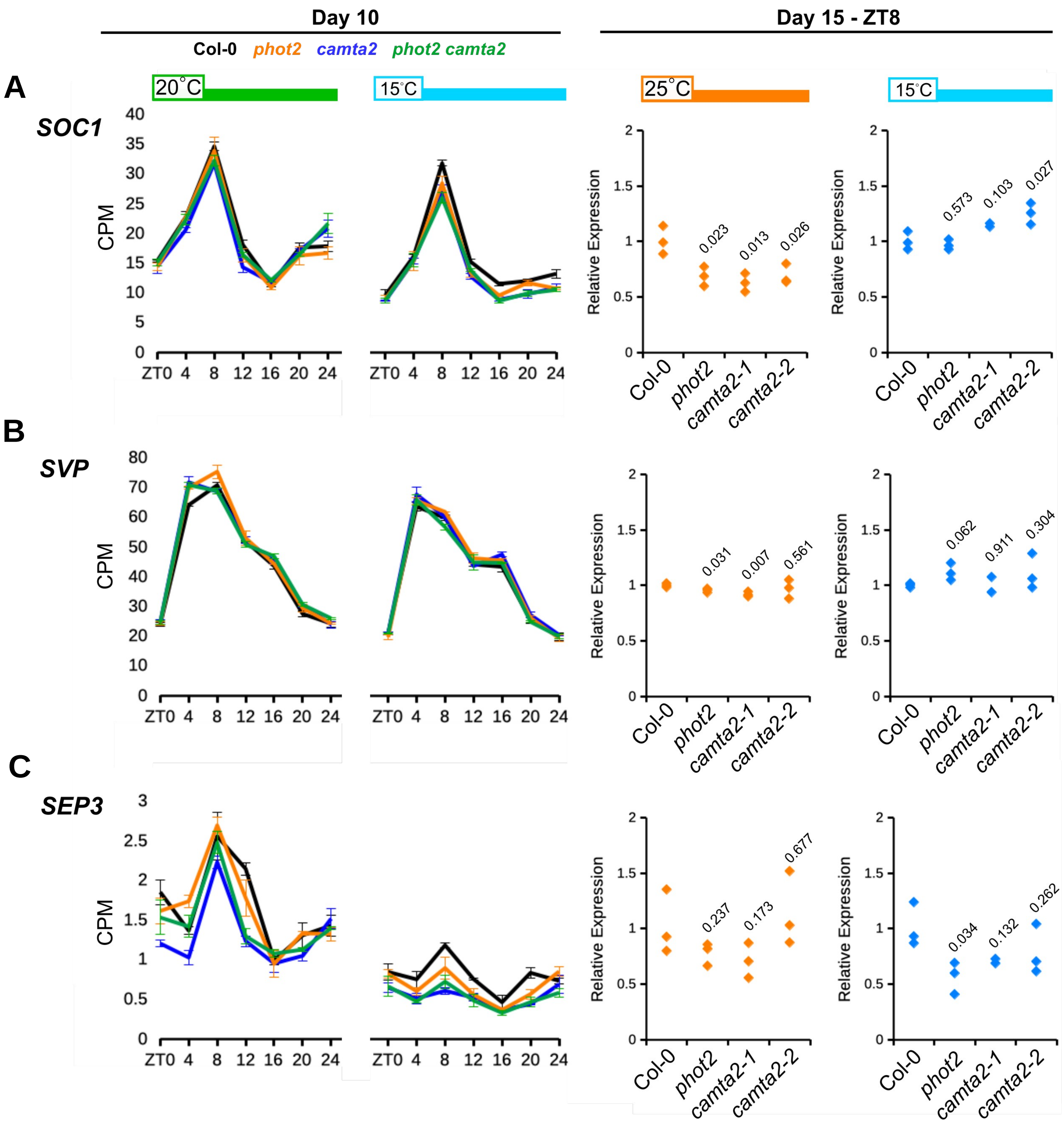

Supplementary Figure 7: Flowering-related gene expression

Expression data for (A) *SOC1*, (B) *SVP*, and (C) *SEP3* from RNAseq on day 10 under 20˚C and 15˚C (time courses at left - Traces are Col-0 (black), p*hot2*(SALK_142275)(orange), *camta2-1*(SALK_007027) (blue), and *phot2 camta2* (green)) and from qRT-PCR at 25˚C and 15˚C (dot plots at right). Color bars at top indicate temperature. T-test (two tailed) p-values for each genotype vs Col-0, within temperature, for qRT-PCR data are noted above data points. qRT-PCR was done in biological triplicate, except for *camta2-1* at 15˚C, which is in duplicate due to a damaged sample.

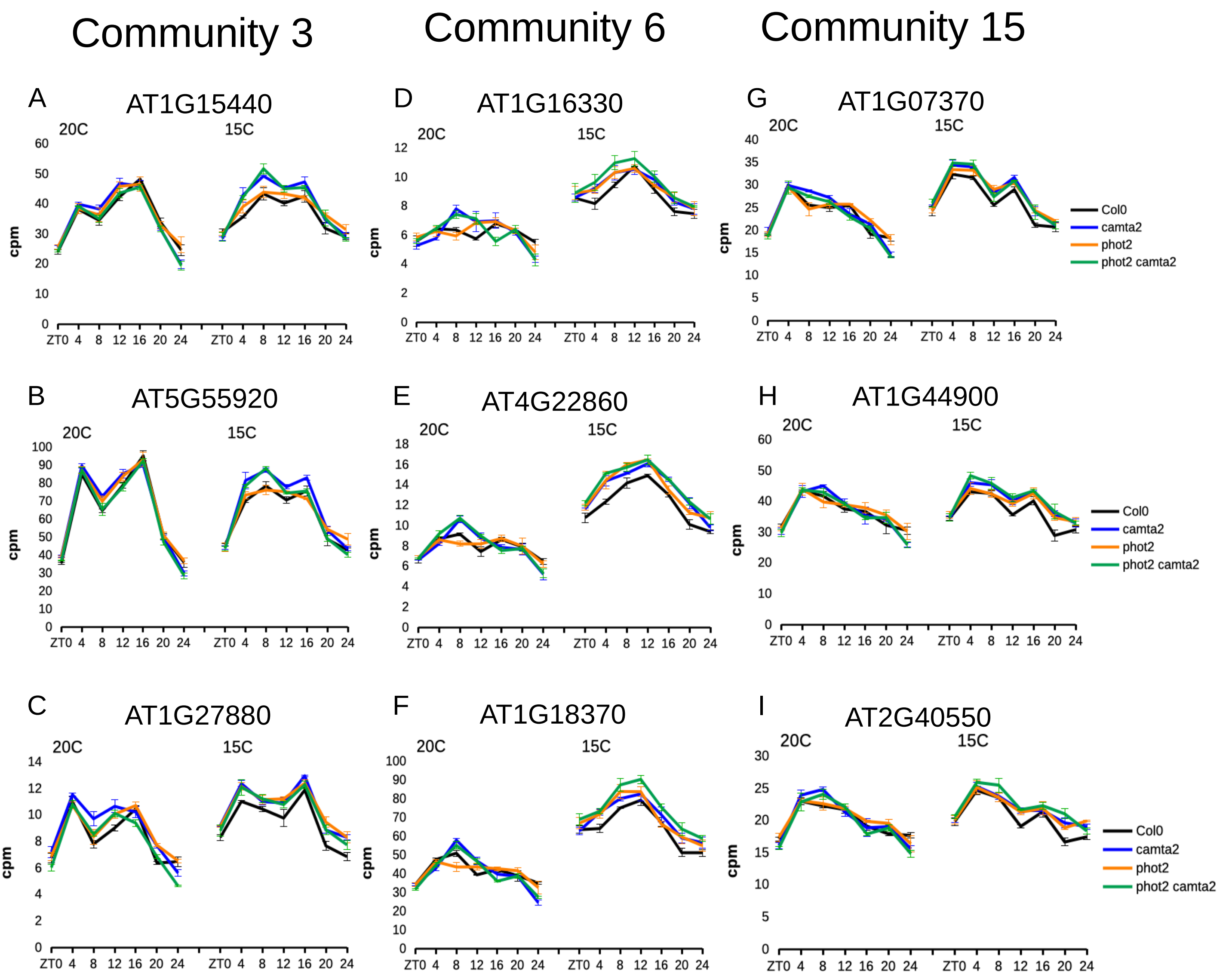

Supplementary Figure 8:
Representative gene expression profiles from communities 3, 6, and 15

(A-C) CPM values of genes in the Ribosome/Translation GO category. (A) AT1G15440, PERIODIC TRYPTOPHAN PROTEIN 2 (PWP2) , (B) AT5G55920, OLIGOCELLULA 2 (OLI2), (C) AT1G27880, DEAD/DEAH-box RNA helicase. (D-F) CPM values of genes within the Cell Cycle GO category. (D) AT1G16330 CYCLIN B3;1, (E) AT4G22860, TPX-like PROTEIN 3 (TPXL3), (F) AT1G18370, NPK1-ACTIVATING KINESIN 1 (NACK1), (G-I) CPM values of genes in the DNA Replication GO category. (G) AT1G07370, PROLIFERATING CELL NUCLEAR ANTIGEN 1 (PCNA1), (H) AT1G44900, MINICHROMOSOME MAINTENANCE 2 (MCM 2), (I) AT2G40550, E2F TARGET GENE 1 (ETG1). Error bars are SEM. Individual data points are not shown for clarity. Traces are Col-0 (black), p*hot2*(SALK_142275)(orange), *camta2-1*(SALK_007027) (blue), and *phot2 camta2* (green).

Community 01

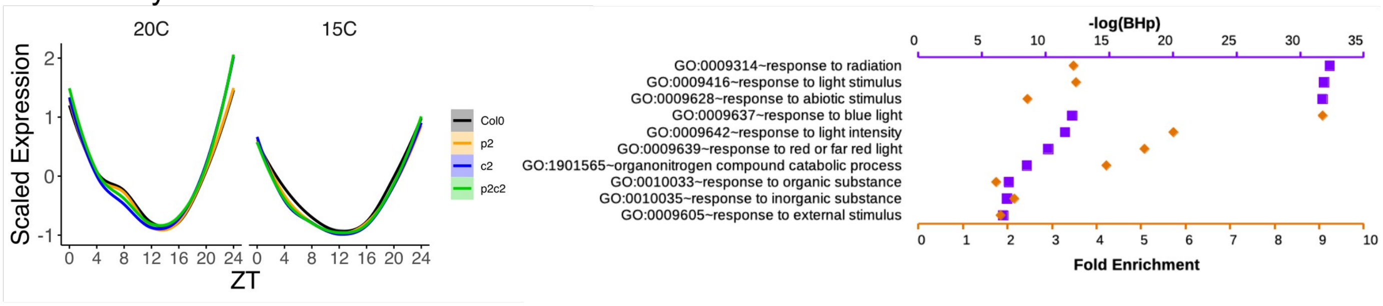

Community 11

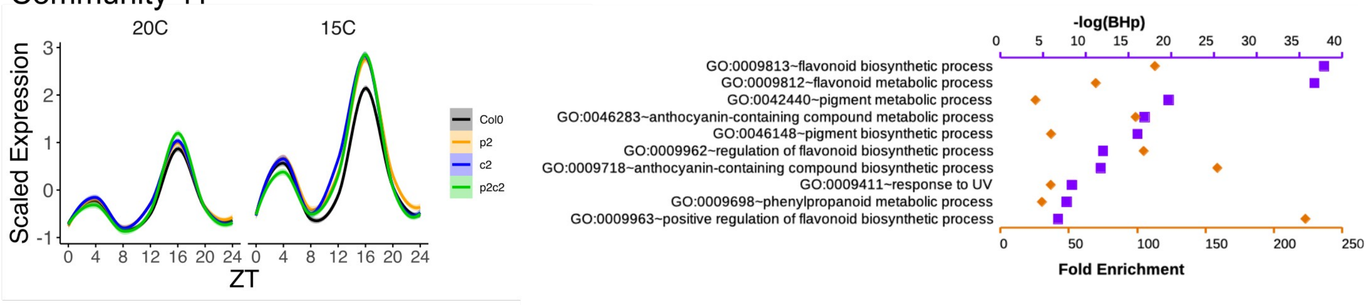

Supplementary Figure 9: Additional gene co-expression communities of interest
Data are presented as in Fig. 4B (mean scaled expression time courses by community), and 4D (Gene Ontology categories, fold enrichment and -log(Benjamini-Hochberg p value)). Traces are Col-0 (black), p*hot2*(SALK_142275)(orange), *camta2-1*(SALK_007027) (blue), and *phot2 camta2* (green).

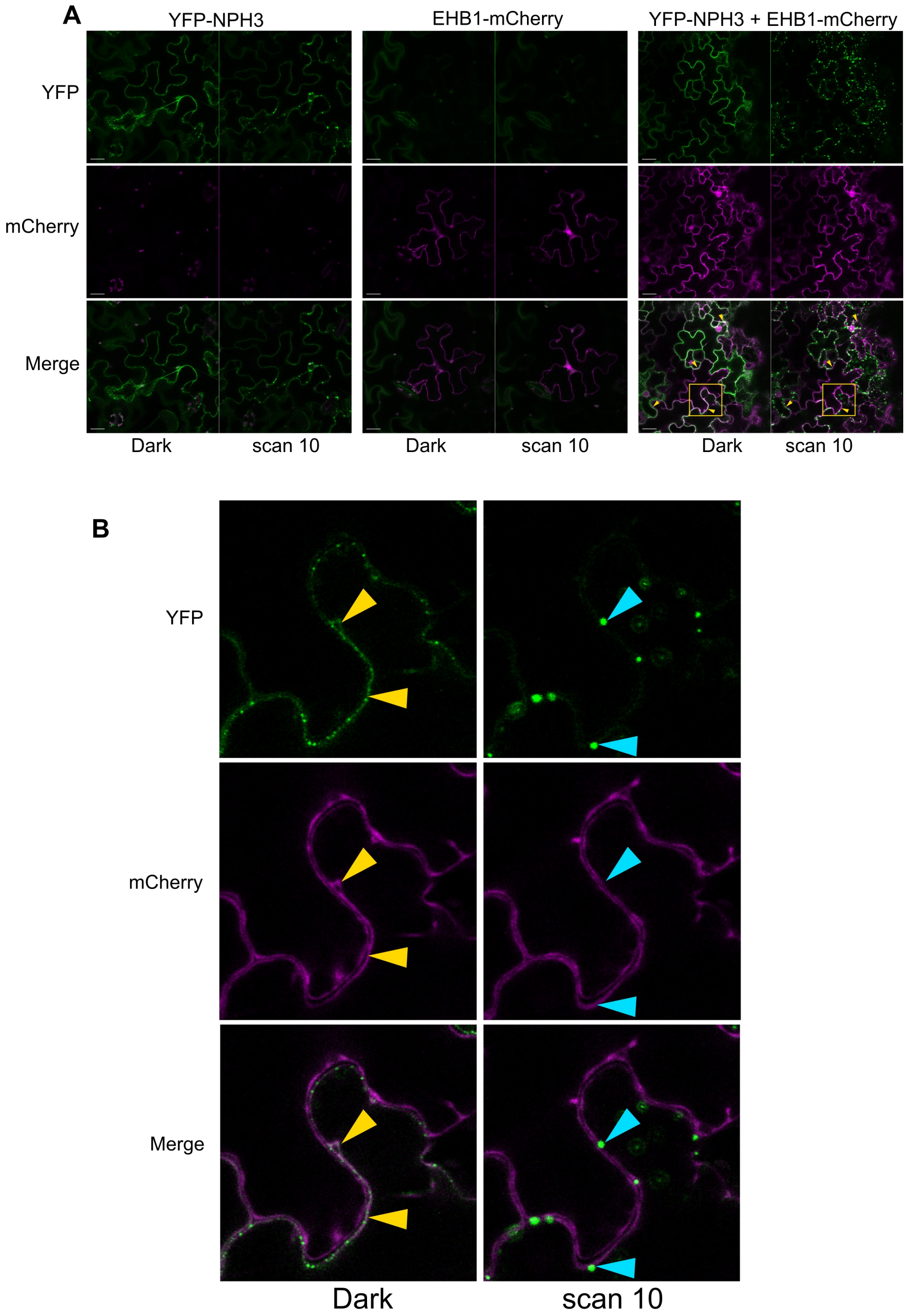

Supplementary Figure 10: Transient co-expression of NPH3 and EHB1 in tobacco
Constructs carrying 35S promoter-driven YFP-NPH3 and EHB1-mCherry were infiltrated into tobacco leaves. Images of the dark adapted (Dark) and 488nm laser exposed (scan 10) samples are shown. Images are representative of three replicates. Confocal images were taken with identical settings. YFP-NPH3: green, EHB1-mCherry: magenta.
(A) Maximum projections of 5 confocal sections. These images were uniformly scaled to a maximum pixel intensity of 128, down from 255, for easier visibility. Scale bar is 20 µm. Unadjusted images are provided in Source Data.
(B) Expanded regions from a single confocal section within the yellow boxes in (A). Yellow arrowheads in Dark frames are regions of potential NPH3-EHB1 co-localization. Blue arrowheads in scan10 frames indicate the position of NPH3 condensates.
