# Supplementary material for: Genetic architecture of a light-temperature coincidence detector
Source: Nat Commun. 2025 Aug 26;16:7947. doi: 10.1038/s41467-025-62194-y (PMC12381198; doi:10.1038/s41467-025-62194-y)
Supplement: Supplementary file 2 — Description of additional Supplementary Files [file 41467_2025_62194_MOESM2_ESM.pdf]

Description of Additional Supplementary Files

Supplementary Data_1_gl1_RNAseq.xlsx

Description: Differentially expressed genes (DEGs) identified in *phot1-5 gl1* and *phot2-1 gl1* plants compared to *gl1* background control.

Supplementary Data_2_TimeCourse_RNAseq.xlsx

Description: DEGs identified in *phot2* (SALK_142275), *camta2-1* (SALK_007027), and *phot2 camta2* vs Col-0. File contains lists of DEGs identified in each mutant at each temperature and time point. Also includes the overlap set of "Common DEGs" that are misregulated in the same direction in all three mutants, as well as the location of expected CAMTA binding motifs in the promoters of these genes.

Supplementary Data_3_Network.xlsx

Description: Contains information on the generation and structure of the co-expression network presented in Figure 4. Includes the input data set in scaled CPM, network connectivity properties, gene descriptions by co-expressed community, enriched gene ontology categories for each community, and two-way ANOVA+TukeyHSD p-values used to generate Figure 4C.

Supplementary Data_4_Primers.xlsx

Description: Contains oligonucleotides and probe sets used for genotyping, cloning, qRT-PCR, and EMSA experiments.
