# Supplementary material for: Genetic architecture of a light-temperature coincidence detector
Source: Nat Commun. 2025 Aug 26;16:7947. doi: 10.1038/s41467-025-62194-y (PMC12381198; doi:10.1038/s41467-025-62194-y)
Supplement: Supplementary file 7 — Reporting Summary [file 41467_2025_62194_MOESM7_ESM.pdf]

nature portfolio

Corresponding author(s): Adam Seluzicki

Last updated by author(s): Jul 1, 2025

Reporting Summary

Nature Portfolio wishes to improve the reproducibility of the work that we publish. This form provides structure for consistency and transparency in reporting. For further information on Nature Portfolio policies, see our Editorial Policies and the Editorial Policy Checklist.

Statistics

For all statistical analyses, confirm that the following items are present in the figure legend, table legend, main text, or Methods section.

n/a	Confirmed	
☐	☒	The exact sample size (*n*) for each experimental group/condition, given as a discrete number and unit of measurement
☒	☐	A statement on whether measurements were taken from distinct samples or whether the same sample was measured repeatedly
☐	☒	The statistical test(s) used AND whether they are one- or two-sided<br>*Only common tests should be described solely by name; describe more complex techniques in the Methods section.*
☒	☐	A description of all covariates tested
☐	☒	A description of any assumptions or corrections, such as tests of normality and adjustment for multiple comparisons
☐	☒	A full description of the statistical parameters including central tendency (e.g. means) or other basic estimates (e.g. regression coefficient) AND variation (e.g. standard deviation) or associated estimates of uncertainty (e.g. confidence intervals)
☐	☒	For null hypothesis testing, the test statistic (e.g. *F*, *t*, *r*) with confidence intervals, effect sizes, degrees of freedom and *P* value noted<br>*Give P values as exact values whenever suitable.*
☒	☐	For Bayesian analysis, information on the choice of priors and Markov chain Monte Carlo settings
☒	☐	For hierarchical and complex designs, identification of the appropriate level for tests and full reporting of outcomes
☐	☒	Estimates of effect sizes (e.g. Cohen's *d*, Pearson's *r*), indicating how they were calculated

Our web collection on statistics for biologists contains articles on many of the points above.

Software and code

Policy information about availability of computer code

Data collection	*Provide a description of all commercial, open source and custom code used to collect the data in this study, specifying the version used OR state that no software was used.*
Data analysis	R 4.1.2<br>edgeR 3.36.0<br>dplyr 1.1.2<br>agricolae 1.3-6<br>igraph 1.4.2<br>reshape2 1.4.4<br>stats 4.1.2

For manuscripts utilizing custom algorithms or software that are central to the research but not yet described in published literature, software must be made available to editors and reviewers. We strongly encourage code deposition in a community repository (e.g. GitHub). See the Nature Portfolio guidelines for submitting code & software for further information.

Data

Policy information about availability of data

All manuscripts must include a data availability statement. This statement should provide the following information, where applicable:

- Accession codes, unique identifiers, or web links for publicly available datasets
- A description of any restrictions on data availability
- For clinical datasets or third party data, please ensure that the statement adheres to our policy

All data are included in the main figures, extended data, supplementary data, and source data. RNA seq data files have been deposited in the NCBI Gene Expression Omnibus (GSE240124).

Research involving human participants, their data, or biological material

Policy information about studies with human participants or human data. See also policy information about sex, gender (identity/presentation), and sexual orientation and race, ethnicity and racism.

Reporting on sex and gender	n/a
Reporting on race, ethnicity, or other socially relevant groupings	n/a
Population characteristics	n/a
Recruitment	n/a
Ethics oversight	n/a

Note that full information on the approval of the study protocol must also be provided in the manuscript.

Field-specific reporting

Please select the one below that is the best fit for your research. If you are not sure, read the appropriate sections before making your selection.

☒ Life sciences ☐ Behavioural & social sciences ☐ Ecological, evolutionary & environmental sciences

For a reference copy of the document with all sections, see nature.com/documents/nr-reporting-summary-flat.pdf

Life sciences study design

All studies must disclose on these points even when the disclosure is negative.

Sample size	Sample sizes were typically limited by space in experimental chambers, and were not pre-determined by statistical methods.
Data exclusions	One RNAseq sample (AS025 - Col-0, 15C, ZT12) was excluded from analysis due to evidence of infection.
Replication	RNAseq data was collected from two (gl1 background) or three (time course) biological replicates with. qRT-PCR experiments contain three biological replicates with the exception of one lost sample. Flowering time was determined for 12-48 individuals per genotype per condition (labeled in figures). Mobility shift assay was repeated twice.
Randomization	RNA-seq: Plants were selected from several parts of the tray and pooled. Flowering assays: Plants were regularly shifted and rotated within experimental chambers to limit position effects.
Blinding	Blinding was not used in data collection or analysis.

Reporting for specific materials, systems and methods

We require information from authors about some types of materials, experimental systems and methods used in many studies. Here, indicate whether each material, system or method listed is relevant to your study. If you are not sure if a list item applies to your research, read the appropriate section before selecting a response.

Materials & experimental systems

n/a	Involved in the study
☐	☒ Antibodies
☒	☐ Eukaryotic cell lines
☒	☐ Palaeontology and archaeology
☒	☐ Animals and other organisms
☒	☐ Clinical data
☒	☐ Dual use research of concern
☐	☒ Plants

Methods

n/a	Involved in the study
☒	☐ ChIP-seq
☒	☐ Flow cytometry
☒	☐ MRI-based neuroimaging

Antibodies

Antibodies used	Monoclonal ANTI-FLAG® M2-Peroxidase (HRP) antibody produced in mouse, clone M2 (Sigma # A8592). Used at 1:2000 dilution.
Validation	https://www.sigmaaldrich.com/specification-sheets/161/592/A8592-BULK________SIGMA____.pdf<br>See also Fig.5C this manuscript: Unprogrammed WGE shows no signal.

Dual use research of concern

Policy information about dual use research of concern

Hazards

Could the accidental, deliberate or reckless misuse of agents or technologies generated in the work, or the application of information presented in the manuscript, pose a threat to:

No	Yes
☒	☐ Public health
☒	☐ National security
☒	☐ Crops and/or livestock
☒	☐ Ecosystems
☒	☐ Any other significant area

Experiments of concern

Does the work involve any of these experiments of concern:

No	Yes
☒	☐ Demonstrate how to render a vaccine ineffective
☒	☐ Confer resistance to therapeutically useful antibiotics or antiviral agents
☒	☐ Enhance the virulence of a pathogen or render a nonpathogen virulent
☒	☐ Increase transmissibility of a pathogen
☒	☐ Alter the host range of a pathogen
☒	☐ Enable evasion of diagnostic/detection modalities
☒	☐ Enable the weaponization of a biological agent or toxin
☒	☐ Any other potentially harmful combination of experiments and agents

Plants

Seed stocks	Seed stocks were obtained from ABRC: SALK_146058, SALK_142275, SALK_110039, SALK_129128C, WISCDsLOX457-490P9, SALKseq_043678.1, SALK_008187, SALK_007027, SALK_139582, SALK_001152, SAIL_385_C07, SAIL_1307_C10.v1
Novel plant genotypes	Mutations were combined using standard genetic crosses and confirmed by PCR.
Authentication	Seed stocks were confirmed using PCR genotyping, and separated from known co-segregating background insertions as reported on the respective stock information pages of ABRC.
